# The pathophysiology of cognitive impairment in individuals with heart failure: a systematic review

**DOI:** 10.3389/fcvm.2023.1181979

**Published:** 2023-05-23

**Authors:** Reine Sam Shi Ni, Hanis Qarissa Mohamed Raffi, Yanhong Dong

**Affiliations:** Alice Lee Centre for Nursing Studies, Yong Loo Lin School of Medicine, National University of Singapore, Singapore, Singapore

**Keywords:** heart failure, cognitive impairment, pathophysiology, systematic review, dementia

## Abstract

**Introduction:**

Heart Failure and Cognitive Impairment are both on the rise and shown to be interlinked. Despite existing reviews delineating a relationship between heart failure and cognitive impairment, the underlying pathophysiology is not researched in great depth. Current literature proposed varying pathophysiological mechanisms and focused heavily on the prevalence of cognitive impairment and treatment interventions such as cardiac rehabilitation. In view of the limitations of previous reviews, this systematic review summarized the best existing evidence concerning different pathophysiological mechanisms behind cognitive impairment in individuals with heart failure.

**Methods:**

Eight electronic databases including PubMed, Cochrane Library and EMBASE etc., two grey literatures (ProQuest Theses and Dissertations and Mednar) and hand-searching of references were performed using specific criteria regarding population, exposures and outcomes, before duplicate removal and screening using Endnote and Rayyan respectively. JBI critical appraisal tools for non-randomized studies were used for appraisal. Data extraction was performed using two modified forms from JBI Manual for Evidence Synthesis.

**Results:**

Narrative synthesis was performed to summarize the data from 32 studies. There were three main themes—cognitive impairment due to changes in the brain: brain atrophy, alterations in grey matter and white matter, cerebral alterations, pathway or axis changes, neuroinflammation and hippocampal gene changes; cognitive impairment due to changes in the heart or systemic circulation: inflammation, oxidative stress and changes in serum biomarkers or proteins and the riser rhythm; cognitive impairment due to changes in both the brain and the heart, with seven studies obtaining negative results. There are some limitations such as having non-human studies and large numbers of cross-sectional studies etc.

**Discussion:**

Considering the findings, future research should examine the bi-directional relationship between the brain and the heart as most of the existing research is about the effect of the heart on the brain. By understanding the different pathophysiological mechanisms, the management and prognosis of heart failure patients will be ameliorated. Interventions that slow down or even reverse cognitive impairment can be explored so that these two common issues will not add to the already aggravating disease burden.

**Systematic Review Registration:**

This review is registered under PROSPERO. Identifier: CRD42022381359.

## 1. Introduction

Heart Failure (HF) is an illness with structural and functional defects in the myocardium, resulting in failure to perform ventricular filling or ejection. HF can be acute or chronic. There are two major types of HF, namely Heart Failure with preserved ejection fraction (HFpEF) and Heart Failure with reduced ejection fraction (HFrEF). In HFpEF, the EF is usually above 50% with the left-ventricular (LV) cavity volume being normal. In HFrEF, the EF is below 40% and LV cavity is dilated (1). There are four HF classes according to the New York Heart Association (NYHA) (2), which would be discussed later. Around 64.3 million people are diagnosed with HF worldwide, with a surge in the younger population (3). HF brings about many co-morbidities. Common ones include chronic kidney disease, diabetes, and obesity, especially in HFpEF (4). Others include hypertension, atrial fibrillation, and ischemic heart disease (3).

The comorbidity discussed in this review is Cognitive Impairment (CI), which is present in around 40% of HF. Patients with HF typically exhibit CI in domains of memory, attention, processing speed, and executive function. They face issues with self-management and medication adherence (5). They have difficulties learning and concentrating. In HF, CI usually ranges from mild cognitive impairments (MCI) to dementia (6). MCI does not fully disrupt patients' daily activities, but it is an important stage before transiting to dementia, which is a bigger health burden. It is crucial to prevent MCI and the development of dementia (7). Common cognitive tools used includes Mini-Mental State Examination (MMSE) and Montreal Cognitive Assessment (MoCA) (8).

Pathophysiology refers to the study of changes in bodily functions which lead to the development of diseases. It usually involves studying biological processes related to the diseases, such as identifying biological markers and mechanisms that can explain the progress of the disease (9).

Two of the existing systematic reviews were more pertinent to this review. The first one included 66 studies and found that cognitive decline was related to medial temporal lobe atrophy (MTA), reduced cerebral blood flow (CBF) and decrease of grey matter (GM). Some other factors included increased B-type natriuretic peptide (NT-proBNP) and the aforementioned comorbidities—Diabetes Mellitus, obesity and atrial fibrillation. However, it concentrated heavily on imaging, which might have restricted the exploration of other pathophysiological mechanisms, albeit being published recently (10). The second one included 148 studies and reported similar findings—brain atrophy, GM, white matter (WM) and poor CBF. Additionally, it included infarcts and microemboli, which influenced cardiac output (11). However, it only utilized PubMed and was published in 2015 hence the data would not be as extensive and updated. Moreover, it extended to all types of CI but this review restricted to MCI, dementia and Alzheimer's Disease (AD) since they are the common CI linked to HF.

Most systematic reviews did not include grey literature. Despite delineating a relationship between HF and CI, the underlying pathophysiology was not researched in great depth. Current literature proposed varying pathophysiological mechanisms but focused heavily on the prevalence of CI in HF and treatment interventions such as cardiac rehabilitation. In light of the limitations, this review summarized the best existing evidence concerning the different pathophysiological mechanisms behind CI in individuals with Heart Failure. The following question was addressed: What is the pathophysiology of Cognitive Impairment in individuals with Heart Failure? The gaps were addressed in this review to provide an updated overview of all the available literature. As CI has negative impacts on the trajectory of HF patients, it is important to understand the mechanisms that correlate them together.

## 2. Materials and methods

The methodology was referenced from Joanna Briggs Institute (JBI) Reviewer's Manual on systematic reviews of etiology and risk (12). This review followed the Preferred Reporting Items for Systematic Reviews and Meta-Analyses Protocols (PRISMA) guidelines and statement (13) and was registered under PROSPERO CRD42022381359. The PRISMA checklist is attached in Supplementary Appendix S1.

### 2.1. Eligibility criteria

The inclusion criteria were as follows: (1) human individual with HF diagnosis or animal HF model; (2) minimally one cognitive tool or diagnosis of cognitive problem(s) including dementia, AD and MCI for human studies; (3) in peer-reviewed journals; (4) published in English or Chinese; (5) quantitative studies—observational studies which encompass cross-sectional, case-control and cohort studies; 6) published from 1 January 2013 to 31 December 2022. This was set as filter whenever possible. If the full date could not be set, it would be set with the year minimally. The limit of 10 years ensured sufficient data whilst not being outdated. Meanwhile, for the other inclusion criteria, they would come into play during screening. The inclusion of animal models was necessary to avoid missing important results as animals especially mice, have more than 95% of homologous genes as humans and were used in numerous research studies (14).

The exclusion criteria were as follows. Firstly, the study was excluded if CI occurred before HF as it would not illustrate the pathophysiology of CI in HF. Secondly, studies that focused heavily on non-HF conditions were excluded, unless they included sufficient data on HF and the pathophysiology of CI on it. This was to prevent missing out on significant studies that could answer the research question and aim. Thirdly, all non-primary papers such as reviews, reports, commentaries, letters, protocols, clinical guidelines, and case reports were excluded.

### 2.2. Information sources

Eight electronic databases, namely PubMed, Cochrane Library, EMBASE, ProQuest Social Science Premium Collection, Scopus, Web of Science Core Collection, CINAHL Complete and PsycINFO were used. The grey literature databases included ProQuest Theses and Dissertations and Mednar. Hand-searching of the reference list and journals was performed.

### 2.3. Search strategy

The three-step search strategy was adopted (12). The first step was an initial search of PubMed and Embase alongside the analysis of the keywords and subject headings contained in the title and abstract. A second search using those keywords and subject headings was conducted across the databases. The third step was examining the reference lists of selected sources to broaden the search results. We developed the search strategy iteratively and sought the assistance of a medical librarian for refinement. The strategies included keywords and subject headings in relation to Heart Failure, Cognitive Impairment, and pathophysiology, though there were variations between databases in terms of the MESH terms, Emtree terms and specific database subject headings. An example of the PubMed search strategy is illustrated in Table 1 by concepts. The full search strategy is included in Supplementary Appendix S2.

**Table 1:** Pubmed search strategy.

**PubMed Filters:** 2013–2022	#1	(“Heart Failure”[Mesh] OR (“heart failure”[Title/Abstract] OR “cardiac failure”[Title/Abstract] OR “reduced ejection fraction”[Title/Abstract] OR “myocardial dysfunction”[Title/Abstract] OR “systolic dysfunction”[Title/Abstract] OR “diastolic dysfunction”[Title/Abstract] OR “heart decompensation”[Title/Abstract] OR “myocardial failure”[Title/Abstract] OR “cardiac decompensation”[Title/Abstract] OR “congestive heart failure”[Title/Abstract])) AND (“Cognitive Dysfunction”[Mesh] OR “Dementia”[Mesh] OR (“cognitive impairment*”[Title/Abstract] OR “cognitive problem*”[Title/Abstract] OR “cognitive dysregulation”[Title/Abstract] OR “cognitive dysfunction”[Title/Abstract] OR “cognitive disorder*”[Title/Abstract] OR “cognitive defect*”[Title/Abstract] OR neurodegenerat*[Title/Abstract] OR dement*[Title/Abstract] OR “cognitive derangement”[Title/Abstract] OR “cognitive decline”[Title/Abstract] OR “mental deterioration”[Title/Abstract] OR “mild neurocognitive disorder*”[Title/Abstract] OR Alzheimer*[Title/Abstract] OR “brain impairment*”[Title/Abstract] OR “brain problem*”[Title/Abstract] OR “brain dysregulation”[Title/Abstract] OR “brain dysfunction”[Title/Abstract] OR “brain disorder*”[Title/Abstract] OR “brain defect*”[Title/Abstract] OR “brain decline”[Title/Abstract] OR “brain derangement”[Title/Abstract])) AND (“Causality”[Mesh] OR “physiopathology”[Subheading] OR “etiology”[Subheading] OR (caus*[Title/Abstract] OR pathophysiology[Title/Abstract] OR physiopathology[Title/Abstract] OR reason*[Title/Abstract] OR “heart-brain connection*”[Title/Abstract] OR “heart-brain”[Title/Abstract] OR etiology[Title/Abstract] OR aetiology[Title/Abstract]))

### 2.4. Selection process

Endnote was used to manage the citations. It processes papers and citations in line with PRISMA (15, 16). The exported records were inclusive of a pre-searched filter—year of publication. Duplicates were removed before exporting to Rayyan. Rayyan allows for proper labelling and independent screening with other reviewers (16). Duplicates were removed again in Rayyan. Citation, title, and abstract screening were performed followed by full-text screening. The selection process and results were reported in the PRISMA chart. The search strategy and study selection were conducted independently by two reviewers (RS and HQ) with a consensus reached among them. The screening tool was piloted for five records to ensure mutual understanding of the criteria. The tool is attached in Supplementary Appendix S3.

For citation, title, and abstract screening, these eligibility criteria were used: (1) related to HF and CI; (2) from January 2013 to December 2022; (3) primary studies; (4) in English or Chinese; (5) non-intervention type [e.g., not randomized controlled trials (RCTs)]; (6) discuss the pathophysiology of CI in HF; (7) quantitative designs. For full-text screening, these eligibility criteria were used: (1) studies that fully fit aforementioned eligibility criteria; (2) access to the full-text. The excluded reports were tagged with the following reasons: (1) non primary study—study is related to HF and CI but is non primary; (2) intervention type study—study is related to HF and CI but is focused on intervention(s) or screening tools; (3) not HF and CI—study is not related to both HF and CI; (4) language—study is not in English or Chinese; (5) HF occurred after CI—different from the eligibility criteria required which was for individuals to have HF before CI; (6) not related to pathophysiology—study is related to both HF and CI but not on pathophysiological mechanisms; (7) year—study is published in a different year compared to the criteria; (8) no access—full-text cannot be accessed, the reports under this tag were included under “Reports not retrieved” in the PRISMA chart.

As Rayyan has “Excluded”, “Maybe” and “Included” labels, the records that passed the citation, title, and abstract screening stage were placed under “Maybe” and assessed subsequently during full-text screening. Besides Rayyan, an excel sheet was used to include the papers that were “Excluded” and “Maybe” during citation, title, and abstract screening. Details such as the title, database and reasons for exclusion were input. This acted as a backup record to prevent missing data. The reports in the excel sheet were indicated as “Excluded” or “Included” eventually after full-text screening.

### 2.5. Methodological appraisal

The JBI checklists were used to appraise the studies by assessing the trustworthiness, relevance, and results. There are three different checklists for case-control, cohort, and cross-sectional studies respectively. They were answered using Yes, No, Unclear and Not Applicable. Eventually, a conclusion was made on the quality of the report and whether to include, exclude or to seek further information (17). The entire assessment was done by two reviewers (RS and HQ) with discussions performed for disagreements. The checklists are attached in Supplementary Appendix S4.

### 2.6. Data extraction

Two independent reviewers (RS and HQ) conducted data extraction for the included studies independently. In the event of disagreements, discussions were done. The data were extracted using forms modified from the guidelines of the JBI Manual for Evidence Synthesis, which were piloted on five studies before usage (12). There were two forms, one for human and one for animal studies since different forms of data would be extracted. These are attached in Supplementary Appendix S5.

For human studies, there were seven main sections to fill up in the form, namely General study details, Participant characteristics, Methods, Study result(s) or outcome(s), Data analysis methods, Others and Reviewer's comments. For animal studies, these seven sections were included with minor alterations. For Participant Characteristics, the age range, type of HF and cardiac function grades were omitted since these were mostly unavailable. For Methods, setting and preparation done were used instead of recruitment methods.

### 2.7. Data analyses

For data analysis, narrative summary was performed whereby the studies were discussed in textual form individually and in groups when there were similarities between the studies (18). This was due to unstandardized cognitive tools used and plenty of varying mechanisms. The studies were first placed in the main themes to discuss as a group due to similarities and those that did not fit were discussed separately.

## 3. Results

**Figure 1:**
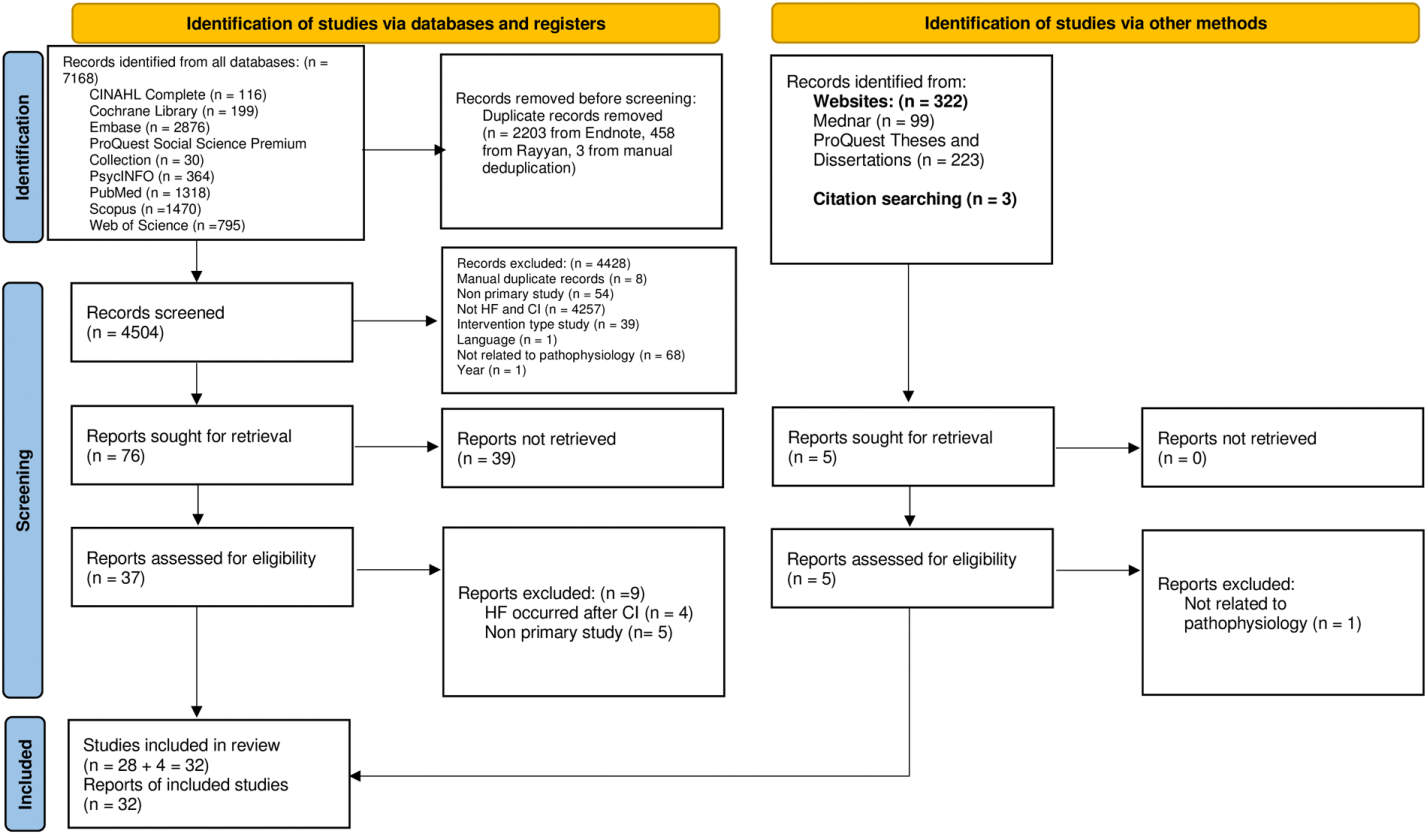
PRISMA chart.

A total of 7,815 records were identified from all databases. After duplicate removal, 5,151 records were set for screening. After citation, title, and abstract screening, there were 81 reports, with 39 being unretrievable. Out of the 39 reports, 37 reports were conference abstracts. Two were not conference abstracts and hence, their full texts were requested. However, one had the abstract only while the other received no response. The remaining 42 reports underwent full-text screening, with 32 studies being eligible. The final studies included 4,630 humans, 452 animals and six datasets. The PRISMA chart is illustrated in Figure 1.

### 3.1. Summary of individual study

The study characteristics are described in Tables 2–5. The study sizes ranged from 37 to 939 for human and nine to 209 for animal studies. The age ranged from 51.4 to 85 years for human and 10 weeks to 10 months for animal studies, with almost all studies reporting in mean and standard deviation. All human studies had more males than females except one cohort study measuring NT-proBNP. Likewise, seven animal studies had male models or more males compared to three with female models or more females involved (19). For human studies, nine studies indicated the type of HF recruited, with the majority being chronic HF (*n* = 9). More studies had non-ischemic causes of HF as opposed to ischemic causes. Some studies included the EF, with a few of them mentioning the mean only whilst the rest mentioning the percentage of HFrEF and HFpEF respectively. Out of those that mentioned the specific type of EF, most of them included more HFrEF than HFpEF, except one study that included more HFpEF instead. The NYHA class were mostly class II with 14 studies, followed by class III with 13 studies and classes I and IV with six studies each. Most studies recruited HF patients in more than one NYHA class, except one that only included class II. Seven studies did not limit the class type. For the case-control human studies, seven studies included healthy controls. Meanwhile, for animal studies, sham groups were used more often than control groups. One human study investigated different illnesses besides HF, namely vascular cognitive impairment (VCI) and carotid occlusive disease (COD), but still focused on HF hence it was included (20). The studies were grouped into three main themes—CI due to changes in the brain in HF: brain atrophy, alterations in GM and WM, cerebral alterations, pathway or axis changes, neuroinflammation and hippocampal gene changes; CI due to changes in the heart or systemic circulation in HF: inflammation, oxidative stress and changes in serum biomarkers or proteins and the riser rhythm; CI due to changes in both the brain and the heart in HF: combination of mechanisms from the previous two sections, with six studies obtaining negative results. The last study with negative results revealed the lack of relationship between AD and HF.

**Table 2:** CI due to changes in the brain in HF.

Authors	Study design	Participants	Diagnosis and type of HF/Cardiac function grade	Cognitive tool(s)	Tool(s) to measure mechanism(s)	Main findings
**Meguro et al., 2017** (21)	Case-control	**37 participants** **HF patients** *N* = 20 Age (median/interquartile range): 76/66–81 years Gender: 7 females/13 males **Controls** *N* = 17 Age: 72/69–80 years Gender: 11 females/6 males	- Diagnosed according to the Framingham criteria - Etiology: 3 ischemic/14 non-ischemic - EF: mean of 46.1% - New York Heart Association (NYHA) Class II	Mini-Mental State Examination (MMSE)	1.5 T MRI scanner and Voxel-based specific regional analysis system for Alzheimer's disease	Local and total brain atrophy (TBA) in the parahippocampal gyrus was prominent and higher in HF patients.
**Frey et al., 2018** (22)	Case-control	**432 participants** **HF patients** *N* = 148 Age: 64 ± 11 years Gender: 23 females/125 males **Controls** *N* = 284 Age: 65 ± 10 years Gender: 44 females/240 males	- Diagnosed according to the guidelines of the European Society of Cardiology - Chronic HF - Etiology: 96 ischemic/52 non-ischemic - EF: mean of 25 less than 35%/mean of 58 between 35% to 44%/mean of 65 more than or equals to 45% - NYHA class I (41) & II (88) & III (19)	Neuropsychological test battery, MMSE	3-T MRI scanner	HF patients exhibited selective deficits in the domains of attention and verbal memory, and medial temporal lobe atrophy (MTA) was identified as a probable structural correlate of cognitive impairment.
**Alosco et al., 2013** (23)	Cross-sectional	**69 HF patients** Age: 68.55 ± 8.07 years Gender: 25 (36.2%) females/44 (63.8%) male	- Diagnosed according to NYHA - Chronic HF - EF: mean of 42.39% - NYHA class II & III	MMSE, American National Adult Reading Test (AMNART)	1.5 T MRI scanner	Greater WMH was independently associated with reduced global cognitive status in HF patients.
					Transcranial doppler (TCD) ultrasonography	Lower TCD measured cerebral perfusion of the middle cerebral artery (MCA) was associated with reduced global cognitive status.
**Alosco et al., 2014** (24)	Cross-sectional	**100 HF patients** Age: 69.49 ± 9.56 years Gender: 31 (31%) females/69 (69%) males	- Diagnosed according to NYHA - EF: 40.6% HFrEF/59.4% HFpEF - NYHA class II (84%) & III (16%)	MMSE, Neuropsychological test battery including TMT A and B, Frontal Assessment Battery (FAB), California Verbal Learning Test-Second Edition	TCD ultrasonography	Reduced cerebral blood flow and subsequent ischemia predicted decreased attention/executive function and memory abilities at the 1-year follow-up.
**Roy et al., 2017** (25)	Case-control	**48 participants** **HF patients** *N* = 19 Age: 55.5 ± 9.1 years Gender: 13 males/6 females **Controls** *N* = 29 Age: 51.4 ± 5.3 years Gender: 18 males/11 females	- Diagnosed according to NYHA - Etiology: 9 ischemic/10 non-ischemic - EF: 100% HFrEF - NYHA class II (80%) & III (20%)	Montreal Cognitive Assessment (MoCA)	3-T MRI scanner	- Regional CBF reduction in HF patients appeared in multiple brain sites, which included vascular beds over the frontal, parietal, and occipital cortices, hippocampus, thalamus, and cerebellar areas. - Reduced CBF in white matter regions are associated with poorer cognitive function.
**Suzuki et al., 2016** (26)	Cross-sectional	**80 participants** **HF patients (Stage C)** *N* = 40 Age: 66.8 ± 8.9 years Gender: 13 (32.5%) females/27 (67.5%) males **Controls (Stage B)** *N* = 40 Age: 65.0 ± 10.9 years Gender: 9 (22.5%) females/31 (77.5%) males	- Diagnosed according to ESC/ACC/AHA guidelines - Chronic HF - Etiology: 43 ischemic/37 non-ischemic - EF: mean for Stage B is 59.6%/mean for Stage C is 43.1%	MMSE, Wechsler Memory Scale-revised	1.5 T MRI scanner	CBF in the posterior hippocampus was lower in Chronic HF patients with Stage C as compared with those with Stage B, and that there was a significant association between CBF in the posterior hippocampus and the extent of cognitive impairment in Chronic HF patients with Stage C.
**Leeuwis et al., 2020** (20)	Cohort	**439 participants** **HF patients** *N* = 124 Age: 68.7 ± 9.9 years Gender: 40 (32%) females/84 (68%) males **Carotid Occlusive Disease (COD) patients** *N* = 75 Age: 65.1 ± 7.5 years Gender: 20 (26.7%) females/55 (73.3%) males **Vascular Cognitive Impairment (VCI) patients** *N* = 127 Age: 68.3 ± 8.7 years Gender: 50 (39.4%) females/77 (60.6%) males **Reference patients** *N* = 113 Age: 65.6 ± 7.1 years Gender: 55 (48.7%) females/58 (51.3%) males	- Diagnosed according to the guidelines of the European Society of Cardiology	MMSE, neuropsychological test battery including Visual Association Test (VAT), Rey Auditory Verbal Learning Test (RAVLT), TMT A and B	3-T MRI scanner	There is reduced whole-brain and regional CBF values in patients with COD and possible VCI compared to patients with HF and reference participants.
**Nyul-Toth et al., 2022** (27)	Case-control	**9 C57BL/6 mice** - 3 bilateral external and internal jugular vein ligation - 6 sham-operated controls **Age:** 10 months **Gender:** Male	- Animal model	Daily Neurological Examination	Histological analysis, Image J software	Cerebral venous congestion exacerbates the genesis of CMHs in a validated mouse model of increased cerebral venous pressure.
**Fulop et al., 2019** (28)	Case-control	**40 C57BL/6 mice** Age: 10 months Gender: Male 1. Bilateral external and internal jugular vein ligation (JVL) 2. Sham-operated control	- Animal model	Neurological Examination, Radial Arm Water Maze	Immunofluorescent labelling and confocal microscopy	Cerebral venous congestion *per se* promotes BBB disruption, which associate with CI in a validated mouse model of increased cerebral venous pressure.
					Quantitative real-time PCR	Cerebral venous congestion *per se* promotes neuroinflammation in the hippocampus, which associate with CI in a validated mouse model of increased cerebral venous pressure.
**Yang et al., 2020** (29)	Case-control	**39 Sprague-Dawley rats (weight of 240± 10 g)** Gender: Male - 19 HF model rats - 20 sham control rats	- Animal model	Morris Water Maze (MWM)	PET imaging, Nissl staining, Transmission Electron Microscopy	Disorders of brain energy metabolism and neuronal structure may be main causes in producing cognitive impairment after heart failure.
**Ichijo et al., 2020** (30)	Cross-sectional	**63 participants** **HF patients** *N* = 35 Age: 70.6 ± 8.8 years Gender: 21 (60%) males/14 (40%) females **Controls** *N* = 28 Age: 70.5 ± 9.3 years Gender: 22 (78.6%) males/6 (21.4%) females	- Diagnosed according to ESC/ACC/AHA guidelines - Etiology: 17 ischemic/18 non-ischemic - EF: 24 HFrEF/11 HFpEF - NYHA class I (21) & II (14)	MMSE, Verbal Fluency Test (VFT)	VFT, Near-Infrared Spectroscopy (NIRS)	Frontal and temporal brain activity (an increase in cerebral oxyhemoglobin concentration in the frontal region and temporal lobes in response to the VFT) and cognitive function (MMSE) were lower in the patients with HF compared with the control subjects.
**Ferro et al., 2020** (31)	Cohort	**278 participants** **HF patients** *N* = 154 Age: 69.5 ± 10.1 years Gender: 49 (32%) females/105 (68%) males **Reference patients** *N* = 124 Age: 65.6 ± 7.4 years Gender: 58 (47%) females/66 (53%) males	- Diagnosed according to the guidelines of the European Society of Cardiology - Etiology: 16 ischemic/10 non-ischemic - EF: 13 HFrEF/2 HFpEF/9 HF mid-range EF	MMSE, neuropsychological test battery	3-T MRI scanner	Among patients with HF, presence of cerebral cortical microinfarcts (CMIs) did not relate to cognitive impairment.
**Toledo et al., 2019** (32)	Case-control	**12 Sprague-Dawley rats (∼250 g)** Gender: Male - 6 HF - 6 Sham	- Animal model	MWM, Memory Flexibility test	Immunoblots	CI in HF rats was correlated to altered Wnt/β-catenin pathway evident by a decrease in β-catenin and phospho-Ser-9 GSK-3β. Alterations in Wnt/β-catenin pathway in the hippocampus are independent of blood perfusion to the brain.
**Feng et al., 2021** (33)	Case-control	- GSE120584 dataset: serum miRNA and clinical data of 1,569 cases of cognitive impairment patients and normal controls - GSE116,250 dataset: mRNA expression data of 64 HF patients and normal controls - GSE140831 dataset: mRNA expression data of 1,129 patients with cognitive impairment and normal controls - RT-PCR data and clinical data from 95 patients served as a test set	NA	NA	Functional Enrichment Analysis, GSEA software	The hsa-miR-933/RELB/CCL21 regulatory axis was considered a potential culprit in the development of both HF and cognitive disorders.

**Table 3:** CI due to changes in the heart or systemic circulation in HF.

Authors	Study design	Participants	Diagnosis and type of HF/Cardiac function grade	Cognitive tool(s)	Tool(s) to measure mechanism(s)	Main results
**Athilingam et al., 2013** (34)	Cross-sectional	**38 HF patients** Age: 61.7 ± 8.8 years Gender: 26 (68.4%) males/12 (31.6%) females	- Diagnosed according to the International Statistical Classification of Diseases and Related Health Problems–Ninth Revision [ICD-9] codes - Etiology: 45% ischemic/55% non-ischemic - EF: 79% HFrEF/21% HFpEF - NYHA class I (4/10.5%) & II (20/52.6%) & III (14/36.8%)	MoCA	ELISA assay kits	The hypothesis that inflammatory processes (as evidenced by elevated levels of cytokines and CRP) may be associated with severity of HF and cognitive function (MoCA score) was not supported in this study.
**Niu et al., 2020** (35)	Cross-sectional	**192 HF patients** Age: 66.1 ± 10.6 years Gender: 121 (63%) males/71 (37%) females	- Diagnosed according to the clinical criteria of Chinese Heart Failure Diagnosis and Treatment Guidelines - Chronic HF - EF: mean of 37.6% - NYHA class II (35/18.2%) & III (106/55.2%) & IV (51/26.6%)	Beijing version of MoCA	Biochemistry analyses	Elevated serum Uric acid (UA) is independently associated with poorer performance on cognitive function in CHF patients, only in male and not female CHF patients.
**Dong et al., 2019** (36)	Cross-sectional	**100 HF patients** **CI** *N* = 42 Age: 64.3 ± 8.3 years Gender: 34 (81%) males/8 (19%) females **No CI** *N* = 54 Age: 54.2 ± 10.1 years Gender: 48 (88.9%) males/6 (11.1%) females	- Diagnosed according to the guidelines of the European Society of Cardiology - Chronic HF - Etiology: 54 ischemic/42 non-ischemic - EF: 61 HFrEF/35 HFpEF - NYHA Class I & II/III & IV → CI: 32 (76.2%)/10 (23.8%) → No CI: 49 (90.7%)/5 (9.3%)	MMSE, MoCA, neuropsychological test battery	Assays	NT-proBNP was independently associated with CI.
**Feola et al., 2013** (37)	Cross-sectional	**303 HF patients** Age: 71.6 ± 9.9 years Gender: 180 (59.4%) males/123 (40.6%) females	- Diagnosed according to NYHA - Congestive HF - NYHA class II (83/27.4%) & III (157/51.8%) & IV (63/20.8%)	MMSE	Fluorescence immunoassay	Plasma BNP inversely correlated significantly with cognitive function (MMSE).
**van Vliet et al., 2014** (19)	Cohort	**560 HF patients** Age: 85 years **Lowest tertile NT-proBNP levels** *N* = 186 Gender: 62 (33%) males/124 (67%) females **Medium tertile NT-proBNP levels** *N* = 187 Gender: 54 (29%) males/133 (71%) females **Highest tertile NT-proBNP** levels *N* = 187 Gender: 71 (38%) males/116 (62%) females	- Diagnosed according to ACC/AHA guidelines - Chronic HF - Etiology: 42.5% ischemic/57.5% non-ischemic - EF: 381 HFrEF/119 HFpEF	MMSE	Roche Modular E-170 automated immunoanalyzer	Higher NT-proBNP levels are associated with worse global cognitive function at baseline in the oldest old.
**Bayes-Genis et al., 2017** (38)	Cohort	**939 HF patients** Age: 66 ± 13.1 years Gender: 682 (72.6%) males/257 (27.4%) females	- Diagnosed according to the guidelines of the European Society of Cardiology - Chronic HF - Etiology: 475 ischemic/464 non-ischemic - EF: 820 HFrEF/119 HFpEF - NYHA class III & IV (213/22.7%); NYHA class I & II (726/77.3%)	Pfeiffer questionnaire	Aβ40 human ELISA Kit	There is no association between bloodstream Aβ40 and cognitive decline observed.
**Komori et al., 2016** (39)	Cross-sectional	**444 participants** **Riser group** *N* = 100 Age: 70 ± 13 years Gender: 55 (55%) males/45 (45%) females **Non-dipper group** *N* = 220 Age: 67 ± 13 years Gender: 138 (62.7%) males/82 (37.3%) females **Dipper group** *N* = 124 Age: 68 ± 12 years Gender: 80 (64.5%) males/44 (35.5%) females	- Diagnosed according to NYHA - Etiology: 138 ischemic/306 non-ischemic - EF: 192 HFrEF/252 HFpEF - NYHA class III & IV: → Riser group: 31% & 31% → Non-dipper group: 49% & 22.7% → Dipper group: 34% & 27.6%	MMSE	Automatic system using electric cuff inflation	Abnormal circadian BP rhythm riser pattern was associated with mild CI in HF patients.

**Table 4:** CI due to changes in both the brain and the heart in HF.

Authors	Study design	Participants	Diagnosis and type of HF/Cardiac function grade	Cognitive tool(s)	Tool(s) to measure mechanism(s)	Main results
**Traub, Otto, Sell, Homola et al., 2022** (40)	Cross-sectional	**146 HF patients** Age: 63.8 ± 10.8 years Gender: 22 (15.1%) females/124 (84.9%) males - 36 Serum glial fibrillary acidic protein (GFAP) < 165 pg/ml - 36 GFAP = 165 to 244.9 pg/ml - 38 GFAP = 245 to 383.9 pg/ml - 36 GFAP ≥384 pg/ml	- Diagnosed according to the guidelines of the European Society of Cardiology - Chronic HF - Etiology: 65% ischemic/35% non-ischemic - NYHA class I (39/26.7%) & II (88/60.3%) & III (19/13%)	Neuropsychological test battery	3-T MRI scanner	GFAP correlated to global brain atrophy, but not beyond ageing.
					Simoa HD-1 analyser instrument	GFAP emerged as an independent predictor of memory dysfunction.
**Traub, Otto, Sell, Göpfert et al., 2022** (41)	Cross-sectional	**146 HF patients** Age: 63.8 ± 10.8 years Gender: 22 (15.1%) females/124 (84.9%) males - 58 with no CI - 88 with CI	- Diagnosed according to the guidelines of the European Society of Cardiology - Chronic HF - Etiology: 65% ischemic/35% non-ischemic - EF: 83.6% HFrEF/16.4% HFpEF - NYHA class II & III	Neuropsychological test battery	3-T MRI scanner	Cerebral and hippocampal atrophy scores were increased.
						Greater WMH volume was found, in association with CI.
					Simoa HD-1 Analyzer instrument	Mild cognitive impairment is associated to subtle increases of pTau and NfL, especially regarding memory function.
**Mueller et al., 2020** (42)	Case-control	**140 participants** **HF patients** *N* = 80 Age: 54.9 ± 5.3 years → *Coronary artery disease and high NT-proBNP levels above 900 pg/ml (CAD) —CAD+* *N* = 35 Gender: 10 females/25 males → *Coronary artery disease and normal NT-proBNP — CAD-* *N* = 23 Gender: 4 females/16 males → *Without CAD — NAD* *N* = 22 Gender: 8 females/14 males **Controls** *N* = 60 Age: 52.2 ± 6.3 years Gender: 17 females/43 males	- Diagnosed according to the guidelines of the European Society of Cardiology	Comprehensive test battery: Trail Making Test (TMT) A, test battery of attentional processes, TMT B, hamasch 5-point test revised, Regensburg word fluency test, standardized Link's probe, California verbal learning test, Rey-Osterrieth complex figure test	3-T MRI scanner	A diminished grey matter density (GMD) was found predominantly in posterior and middle cingulate cortex and precuneus.
					Roche Modular analyzer, Simpson's Method	Decreased ejection fraction and increased NT-proBNP were found in wide brain regions including the whole frontomedian cortex as well as hippocampus and precuneus.
**Kure et al., 2016** (43)	Case-control	**76 participants** **HF patients** *N* = 36 Age: 68 ± 7 years Gender: 25 (69%) males/11 (31%) females **Controls** *N* = 40 Age: 67 ± 5 years Gender: 20 (50%) males/20 (50%) females	- Diagnosed according to NYHA - Etiology: 9 ischemic/27 non-ischemic - NYHA II (30/83%) & III (5/14%) & IV (1/3%)	MMSE, Clinical Dementia Rating (CDR), TMT A and B, Stroop Color Word Test	TCD	Poor attention and executive function abilities in elderly HF patients could be explained by reduced CCA-BFV and MCA-BFV.
					Immunoassay	HF inflammation (high sensitivity C-Reactive Protein) was significantly elevated compared to control group but it was not associated with CI.
					Negative ionization mass spectrometry	The oxidative stress marker, diacron-reactive oxygen metabolites (d-ROMs), was significantly elevated but not associated with CI.
					Chemical Assay	Poor attention and executive function abilities in elderly HF patients could be explained by reduced plasma antioxidant CoQ10 levels.
**Yu et al., 2020** (44)	Case-control	**24 Sprague-Dawley (280–320 g) rats** Age: 10 weeks Gender: Male - 6 sham (Con) - 6 sham + probiotics (CPO) - 6 HF - 6 HF + probiotics (HPO)	- Animal model	MWM, Open Field Test (OFT)	Histopathologic analysis, Image J software	Intestinal flora dysbiosis caused by HF appears to partly aggravate neuroinflammation by impairing the permeability of the BBB and the intestinal barrier.
					Immunofluorescence, Western blot	HF induces gut inflammation and may thus lead to low levels of systemic inflammation and the development of neuroinflammation evident from the increase in pro-inflammatory cytokines in the hippocampus.
**Adamski et al., 2018** (45)	Case-control	**209 participants** **Gender: Female** - 107 Tg*α*q*44 mice → 25 3-month Tgαq*44 → 31 6-month Tgαq*44 → 51 10-month Tgαq*44 **- 102 FVB mice (wild-type control mice)** → 24 3-month FVB →28 6-month FVB → 50 10-month FVB	- Animal model	OFT, Novel Object Recognition (NOR)	Immunohistochemistry, Evans Blue (EB)	The brain endothelial dysfunction was partly evidenced by BBB permeability as well as impairment of endothelial NO-dependent regulation of vascular tone in cortical arterioles.
					Immunoreactivity to E-selectin	The brain endothelial dysfunction was partly evidenced by inflammatory activation.
					Immunohistochemistry	The brain endothelial dysfunction was partly evidenced by increased oxidative stress in the cortex.
						The brain endothelial dysfunction was partly evidenced by β-amyloid cortical accumulation.
**Hong et al., 2013** (46)	Case-control	**49 C57BL/6J mice** Age: 10–14 weeks Gender: 24 males/25 females - 29 HF - 20 sham	- Animal model	OFT, MWM, Elevated Plus-Maze (EPM) test	PET/CT imaging, EB, immunohistochemical staining	BBB was slightly disrupted but it was not a significant result in affecting CI. No difference was found in cerebral glucose metabolism between chronic HF mice and sham mice. Degeneration of neurons was not detected as well.
					Quantitative real-time PCR	Chronic HF and ensuing alterations of cerebral microenvironment led to changes in inflammation-related genes to a different extent in male and female mice and between cerebral cortex, hippocampus and cerebellum. Females have increased expression of inflammatory genes.
					Thioflavin-S staining, immunohistochemical staining, immunoblot	Significant β-amyloid accumulation was not detected. GFAP expression was the same between HF and sham mice in the cortex and hippocampus.
**Kim et al., 2017** (47)	Case-control	**32 Sprague-Dawley rats (250–300 g)** Gender: Male - 8 control - 8 sham - 8 HF - 8 HF plus Losartan group	- Animal model	MWM	Fluorescent probe (MitoSOX)	Ang II induced apoptotic death of cultivated HCNs was due to augmented mitochondrial ROS production.
					Quantitative real-time PCR, Trypan Blue exclusion tests, representative immunoblot	Augmented mitochondrial ROS production activated AMPK-PGC1α signaling.
					Immunoblot	Augmented mitochondrial ROS production led to a increase in proapoptotic protein Bax.
**Baranowski et al., 2021** (48)	Case-control	**9 Ossabaw swine (15 to 20 kg)** Age: 2 months Gender: Female - 5 non-sham sedentary control (CON) - 4 Western Diet-fed aortic-banded (WD-AB) with HF	- Animal model	NA	Immunoprecipitation, Immunoblotting	Cerebrovascular dysfunction was coupled with MAPK pathway activation, specifically neuronal ERK, JNK, p38 signaling and a decrease in basal endothelial NO signaling.
						Cerebrovascular dysfunction was coupled with neuroinflammation.
						Cerebrovascular dysfunction was coupled with amyloidogenic processing indicated by increased APP, BACE1, CTF, and Aβ40 content in the prefrontal cortex and hippocampus of WD-AB swine.
**Islam et al., 2021** (49)	Case-control	**29 mice** Age: 3 months Gender: Male - 16 CamkII*δ*c transgenic of C57Bl/6J mice - 13 control mice (wild type littermates)	- Animal model	OFT and Barnes Maze experiment	Quantitative real-time PCR, Western Blot, Immunoprecipitation	The genes that were up-regulated significantly overlap with genes deregulated in neurons exposed to cellular stress such as oxidative and endoplasmic reticulum (ER) stress, leading to down-regulation of hippocampal genes essential for cognition.
						Hippocampal genes down-regulated in response to HF represent cellular processes linked to cognition and are similar to the gene expression changes observed in models for dementia.

**Table 5:** Relationship between AD and HF.

Authors	Study design	Participants	Diagnosis and type of HF/Cardiac function grade	Cognitive tool(s)	Tool(s) to measure mechanism(s)	Main results
**Duan et al., 2022** (50)	Case-control	**Two genome-wide association study (GWAS) datasets** - 63,926 individuals for GWAS dataset and 10,528,610 single-nucleotide polymorphisms (SNPs) for AD - 977,323 individuals for GWAS dataset and 7,773,021 SNPs for HF - 12 SNPs for bidirectional analysis - 9 SNPs for effect of HF traits on AD	NA	NA	Inverse variance weighted, weighted median and MR-Egger analyses	The study did not support the hypothesis that there is a meaningful causal relationship between genetically predicted HF and AD because the statistical analysis did not provide sufficiently conclusive results to support the hypothesis (*p* > 0.05).

### 3.2. Methodological quality

This review included 32 studies—17 case-control, 11 cross-sectional and four cohort. The summarized appraisal is shown in Supplementary Appendix S6. The results were decided by two reviewers (RS and HQ). All the studies were eventually included though there were studies that had some “No” and “Unclear”, indicating compromised quality. For case-control studies, three studies had no “No” or “Unclear”. The remaining studies had 0–4 “Unclear” and 0–2 “No”, but all studies had minimally six “Yes” out of 10 questions. For cross-sectional studies, three studies had no “No” or “Unclear”. The remaining studies had 0–2 “Unclear” and 0–2 “No”, with all studies having minimally five “Yes” out of eight questions. For cohort studies, all studies had 1–4 “Unclear” and 0–2 “No”, with minimally five “Yes” out of 11 questions.

### 3.3. Study characteristics

#### 3.3.1. CI due to changes in the brain in HF

##### 3.3.1.1. Brain atrophy

Two studies reported on the relationship between CI and brain atrophy. These studies are illustrated in Table 2. One study illustrated local and total brain atrophy (TBA) in the parahippocampal gyrus of HF patients as compared to the controls. Though the severity of TBA was similar between HF and control group, the averaged Z-score which measured the severity of atrophy in parahippocampal gyrus was much higher in HF than control group (21). The other study illustrated more severe MTA and worse attention and memory in HF compared to control group (22). Both utilized magnetic resonance imaging (MRI) to demonstrate the atrophy.

##### 3.3.1.2. Alterations in GM and WM

One study reported on the relationship between CI and GM and WM changes, specifically increased white matter hyperintensities (WMH) (23). It is illustrated in Table 2. Besides the use of MRI, it utilized the Transcranial Doppler (TCD) which allow effective measuring of perfusion. The mean EF was 42.39% which is a mid-range EF, suggesting that of the inclusion of HF with both HFrEF and HFpEF participants.

##### 3.3.1.3. Cerebral changes

10 studies reported on the relationship between CI and cerebral changes. These studies are illustrated in Table 2. Five studies illustrated the relationship between CI and CBF, albeit in different regions. One study reported that reduced CBF velocity (CBFV) of middle cerebral artery (MCA) was related to increased WMH and as aforementioned, increased WMH was related to poorer cognitive function (23). Another study reported reduced CBFV in the posterior cerebral artery (PCA) and middle cerebral artery after a year of follow-up (24). One study reported reduced CBF over the frontal, parietal, and occipital cortices, hippocampus, thalamus, and cerebellar area, with more decline on the right side in cortical and diencephalic areas, indicating laterality in CBF reduction. It was the only study that included fully HFrEF, which might thus illustrate reduced CBF is one of the more definite causes of CI in HF (25). The above three studies all involved NYHA classes II & III. Another study reported reduced CBF in posterior hippocampus for chronic HF participants (26). The last study did not report any association for both whole-brain or regional CBF in HF participants (20). TCD and MRI were the two main tools used to detect CBFV. Two studies illustrated the relationship between CI and cerebral venous congestion. One study reported that cerebral venous congestion led to cerebral microhemorrhages (CMHs) (27) whilst the other stated it caused blood-brain barrier (BBB) disruption instead, leading to CI (28). One study reported that issues with glucose metabolism and structure of neurons contributed to CI (29). The above three studies were all animal models. One other study reported increase in frontal and temporal brain activity, particularly increase in oxyhemoglobin concentration, contributed to CI. There were more participants with non-ischemic causes of HF and HFrEF, it focused more on NYHA classes I and II as well (30). One study reported no association between CI and cerebral cortical microinfarcts (CMI), despite including participants with both ischemic and non-ischemic causes of HF and all ranges of EF (31).

##### 3.3.1.4. Pathway or axis changes

Two studies reported on the relationship between CI and pathway or axis changes. These studies are illustrated in Table 2. One study reported the changes in Wnt/β-catenin pathway in the hippocampus as a contributor (32), whilst the other reported the hsa-miR-933/RELB/CCL21 axis as another contributor (33). They were both case-control studies though one utilized an animal model while the othere was using datasets.

##### 3.3.1.5. Neuroinflammation

One study reported on the relationship between CI and neuroinflammation, specifically evident from increased pro-inflammatory cytokines found in the hippocampus of the animal model (28). It is illustrated in Table 2.

#### 3.3.2. CI due to changes in the heart or systemic circulation in HF

##### 3.3.2.1. Inflammation

One study reported the lack of association between inflammation and CI, despite including participants with both ischemic and non-ischemic causes of HF, all ranges of EF and almost all classes of NYHA. This was the only study that was diagnosed using the International Statistical Classification of Diseases and Related Health Problems–Ninth Revision (34). It is illustrated in Table 3.

##### 3.3.2.2. Changes in Serum biomarkers or proteins

Five studies reported on the relationship between CI and biomarkers or proteins. These studies are illustrated in Table 3. One study reported that increased uric acid (UA) was related to poorer cognition only in male patients. This was the only study that utilized the Beijing version of MoCA and was diagnosed using the Chinese Heart Failure Diagnosis and Treatment Guidelines. It focused on chronic HF but included all forms of etiology of HF and had a mean of 37.6% EF, meaning that most participants were of HFrEF (35). Three studies reported higher NT-proBNP was associated with CI (19, 36, 37). Two studies focused on chronic HF, with one on congestive HF. All three studies included more HFrEF participants and used MMSE as one of the tools to measure cognitive function. One study reported no association between Aβ40 and CI. It included more participants with ischemic etiology, HFrEF and all classes of NYHA. It is the only study to utilize the Pfeiffer questionnaire for cognitive function (38).

##### 3.3.2.3. Riser rhythm

One study reported irregular circadian blood pressure (BP) rhythm being related to MCI. it included more participants with non-ichemic etiology and HFpEF (39). It is illustrated in Table 3. It is a relatively specific mechanism, being the only study that discussed about this. Hence, it requires further research to validate it.

#### 3.3.3. CI due to changes in both the brain and the heart in HF

10 studies reported varying mechanisms involving both the brain and the heart or systemic circulation. Some of the major mechanisms have been mentioned previously, with oxidative stress and hippocampal gene changes being new ones. These are illustrated in Table 4. Four studies were human studies with the remaining six being animal models. The first study illustrated increase in global cerebral atrophy scores in relation to serum glial fibrillary acidic protein (GFAP), which were independently associated with CI (40).

The second study reported increased cerebral and hippocampal atrophy scores. It also illustrated increased WMH, phosphorylated Tau protein (pTau) and neurofilament light chain (NfL), which are key biomarkers of AD (41). These two studies focused on chronic HF, with more participants having ischemic etiology. They both used MRI and the Simoa Analyzer instrument as tools for cognitive function.

The third study reported reduced GM density in specific regions. Additionally, there was reduced EF and increased NT-proBNP in the cortex, hippocampus and precuneus. These were linked to CI (42). The above three studies utilized MRI as a tool for measuring cognitive function.

The fourth study reported that out of the four main outcomes, two were shown to contribute to CI — reduced CBF in middle cerebral artery and common carotid artery (CCA) and reduced antioxidant CoQ10. For the other two outcomes, despite illustrating increase in both oxidative stress and inflammatory markers such as C-Reactive Protein, they were not associated with CI (43)..

The fifth study reported that impaired blood-brain barrier permeability was due to intestinal flora dysbiosis. The gut inflammation led to neuroinflammation as well, evident from increased pro-inflammatory cytokines in the hippocampus. These were contributors to CI (44).

The sixth study reported that four different causes, namely oxidative stress, β-amyloid accumulation, impaired endothelial NO-dependent regulation and inflammation caused endothelial damage, which was linked to CI (45).

The seventh study reported neuroinflammation as a contributor, albeit difference found between gender and brain regions. However, there was slight but insignificant disruption of blood-brain barrier, with glucose metabolism and degeneration of neurons not being contributors of CI. β-amyloid accumulation was not found and GFAP did not contribute to CI as well (46).

The eighth study reported that Angiotensin II induced apoptosis of hippocampal neural stem cells (HCNs) was caused by AMPK-PGC1*α* signaling. This was due to the increase in reactive oxygen species (ROS) which led to concomitant increase of pro-apoptotic Bax protein (47).

The ninth study reported the activation of MAPK pathway and reduced NO signaling as contributors to CI. Other contributors include amyloidogenic processing and thus increased β-amyloid in the prefrontal cortex and hippocampus and neuroinflammation evident from increased ERK, JNK and p38 (48).

The last study reported that hippocampal gene changes led to down-regulation of genes responsible for cognition, which were similar to those observed in animal models with dementia. This was caused by cellular stress such as oxidative and endoplasmic reticulum stress (49).

#### 3.3.4. Relationship between AD and HF

One study reported a lack of association between HF and AD, due to lack of conclusive statistical analysis results (50). This is illustrated in Table 5. It is a case-control study that utilized datasets, similar to another study mentioned previously.

## 4. Discussion

**Figure 2:**
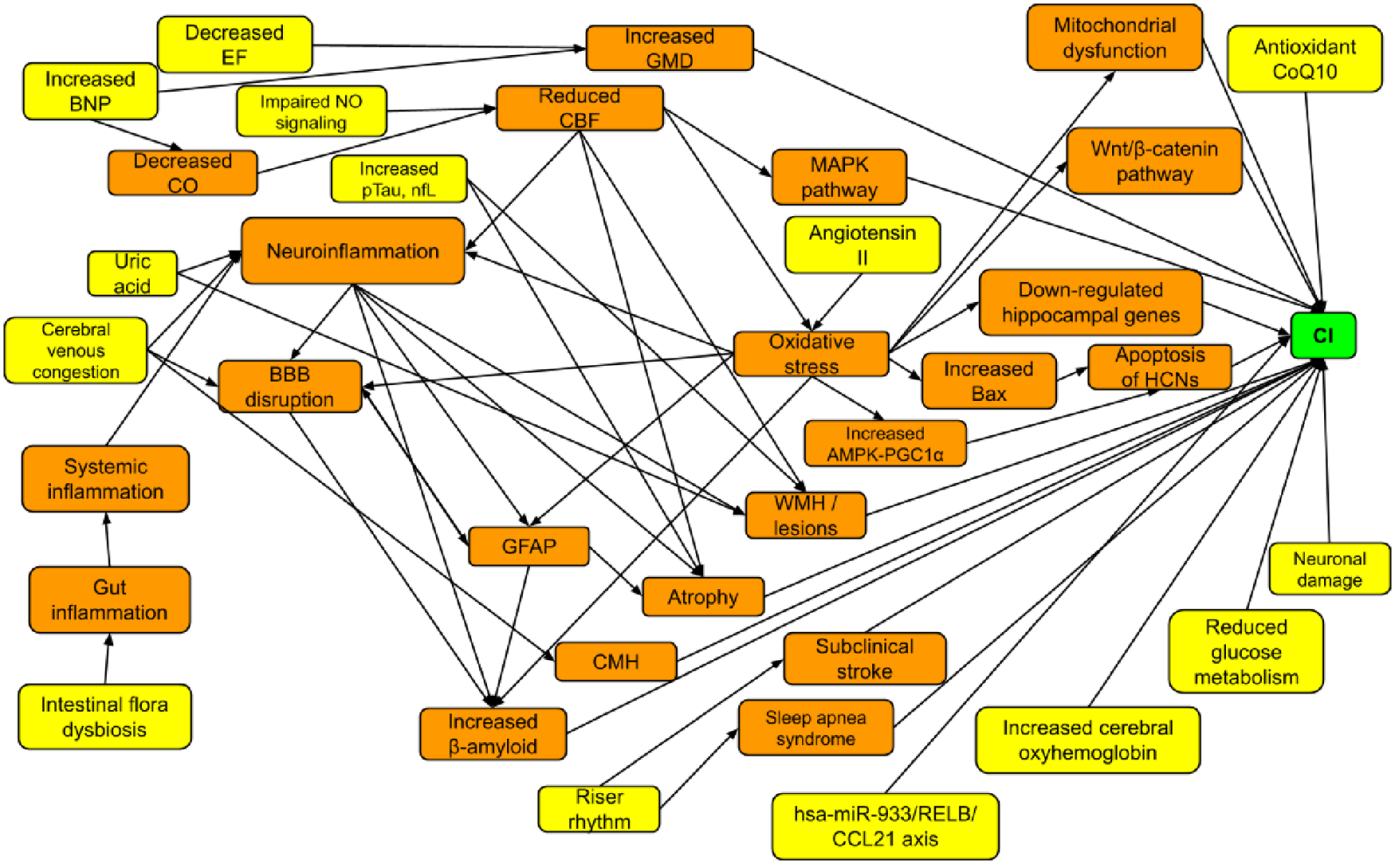
Summarized pathophysiological mechanisms.

With the aim of summarizing the best available evidence concerning different pathophysiological mechanisms behind CI in individuals with Heart Failure, three main themes—CI due to changes in the brain: brain atrophy, alterations in GM and WM, cerebral alterations, pathway or axis changes, neuroinflammation and hippocampal gene changes; CI due to changes in the heart or systemic circulation: inflammation, oxidative stress and changes in serum biomarkers or proteins and the riser rhythm; CI due to changes in both the brain and the heart: combination of some mechanisms from the previous two sections, were identified. Our findings corroborated with both Jiang et al. (10) and Ampadu and Morley (11) as 11 different studies discussed about the same findings in terms of changes in the brain—brain atrophy, changes in GM and WM and decreased CBF. One study produced negative results in terms of atrophy. Possible reasons include some participants using anti-hypertensive medications which might affect the CBF and the delay in transit time during the usage of arterial spin labeling technique. It did not include all potential confounders as well (20). Four studies discussed about NT-proBNP, like Jiang et al. (10). Akin to Ampadu and Morley (11), 10 studies mentioned about oxidative stress, infarcts, and inflammatory cytokines. Ferro et al. (31) concluded that cerebral cortical microinfarcts had no influence on CI, which could be explained by the recruitment of clinically stable HF patients only. Kure et al. (43) concluded that both inflammation and oxidative stress were not statistically significant, possibly due to the small sample size of 36 HF patients, milder HF as 83% were NYHA class II and the exclusion of demented individuals based on MMSE scores. Athilingam et al. (34) proposed negative results on inflammation, possibly due to recruitment of stable HF patients. Being a pilot and cross-sectional study, it might not delineate the causal association well and there was no reference group. Nevertheless, there were new mechanisms that surfaced, such as pathways, genes, antioxidant, electrolyte and protein changes, blood-brain barrier and glucose metabolism issues. Three other reviews done previously had similar findings regarding reduced CBF and inflammation, with Toledo & Andrade et al. (51) discussing about oxidative stress and Wnt signaling as well, Xu et al. (52) on oxidative stress, GFAP and β-amyloid and Goh et al. (53) on atrophy, GM and WM changes. Some of these mechanisms are interlinked and the summary is demonstrated in Figure 2. The yellow coded boxes illustrate the initial causes of CI, with the orange coded boxes being the linkages leading to the green coded CI. Despite the presence of many causes, many pathways are eventually linked to neuroinflammation, oxidative stress, atrophy and increase in β-amyloid.

Besides the aforementioned studies, there were three more studies with negative results. Hong et al. (46) concluded that GFAP, β-amyloid, blood-brain barrier, glucose metabolism and neurodegeneration were insignificant in contributing to CI. This might be attributed to it being an animal study and modeling of mice with myocardial infarction and congestive HF, which was a more specific population type. Bayes-genis et al. (38) concluded that Aβ40 was irrelevant in contributing to CI, possibly due to the usage of a less common cognitive tool—Pfeiffer questionnaire and the loss of nearly half the participants due to death, albeit the study focusing on mortality as an outcome as well. Duan et al. (50) concluded that there was no association between AD and HF, possibly due to restricting to European populations and lack of test set to evaluate the model objectively.

There are both strengths and limitations to this review. For the strengths, they include having two reviewers which helped to reduce inclusion bias. The results were more updated than previous systematic reviews and included grey literature to minimize publication bias, which was lacking in previous systematic reviews. Studies with contradictory results were included to ensure fair representation instead of purely portraying one side and minimized selection bias. Additionally, eight databases were utilized, ensuring more comprehensive search results. There were more variety of study types instead of mostly cross-sectional ones by Jiang et al. (10). For the limitations, this review included non-human studies which might not be fully replicable of the human system and thus there might be information bias. More than a quarter of the studies were cross-sectional hence the causal relationship might not be identifiable and observational studies in general, might bring about more confounders. Additionally, only English and Chinese language publications were included due to reviewers’ inability to understand other languages, leading to publication bias, albeit only one exclusion due to language. A few of the included studies had compromised quality based on the quality appraisal performed, which made the study results less credible.

These findings will be important in informing the healthcare staff on better ways to deal with this comorbidity of HF and to put more focus on it by monitoring and advising HF patients about it. Moving on, more brain imaging can be performed in HF patients as numerous mechanisms involve the changes in CBF and brain structure such as atrophy, amyloid plaques and apoptosis of cells etc. In conjunction with brain imaging, standardized cognitive screening tools can be used as well for more accurate and prompt diagnosis and management plans to be conceived. There are currently many varying tools whereby some may be less effective in the detection of potential CI. Routine blood samplings may involve the testing of significant biomarkers such as UA, NT-proBNP, CRP, phosphorylated Tau protein and ROS metabolites etc. This was noted in Ampadu and Morley (11) as well that there should be screenings to check for CI and rise in NT-proBNP. These increase the probability of noticing any cognitive changes before they develop to irreversible cognitive impairments as these are not checked for routinely. Timely interventions can then be put in place after recognizing the signs of disease progression to prevent further deterioration.

The studies included varying NYHA classes—NYHA classes I to IV. Most of the studies included classes II and III which are the more moderate HF classes. Hence, the pathophysiological mechanisms summarized may be more relevant to those with moderate severity of HF as opposed to the extreme ends. The studies did not specifically illustrate the mechanisms for different classes but discussed as a whole. Most studies included more HFrEF participants, which might suggest that the mechanisms are more relevant to this form of HF. Nevertheless, as the studies still varied in terms of other aspects such as diagnostic tool or etiology or class of HF etc, there were no obvious linkages between these differences and the different pathophysiological mechanisms proposed. Hence, this can be explored further for future studies, to determine the precise mechanisms for different groups of HF participants. More research must be performed to determine suitable tools and treatments for different severity of HF and thus, different severity of CI.

Additionally, an estimated 3.4%–6.7% of hospitalized patients have HF in Asia and around one million people have HF in Japan. HF patients in Asia are mostly younger than in Western countries. However, the youngest in this review was 51.4 years old, thus there was no focus on the younger population *per se*. Hence, future studies can focus on them to determine if there are any differences in the mechanisms depending on age. Thereafter, different tools can be utilized to detect the mechanisms at different ages. In the elderly population, HF conditions are more likely to reduce appetite, decrease muscle mass and physical activity, resulting in malnutrition, frailty and poorer prognosis of HF (54). Groenewegen et al. (3) purported that compared to men, women have lower incidence of HF, albeit them still occupying around half of the prevalent cases. HFpEF is more prevalent in women as they are often more obese than men. In terms of CI, Hong et al. (46) reported worse outcomes in females. However, Niu et al. (35) reported otherwise—increased UA affected only males' cognition.

Future primary research can continue to validate and postulate further pathophysiological mechanisms, especially on those that had contradictory results, albeit being the minority. Understanding the pathophysiology of diseases will allow the effective evaluation of health status and development of suitable treatments and plans (9). Furthermore, the mechanisms are mostly interlinked, hence realizing these linkages can help to prevent further advancement and thereafter deterioration of CI in HF individuals.

## 5. Conclusion

In conclusion, from 32 studies, it is evident that HF and CI are interlinked through the various mechanisms, in terms of changes in the brain and or heart. Future primary research can continue to validate and postulate further pathophysiological mechanisms, especially on those that had contradictory results, albeit being the minority. Larger longitudinal studies will better describe the causal relationship and development of CI in HF, potentially leading to more discovery. Being aware of the pathophysiological mechanisms allows for more pharmacological and non-pharmacological methods to be established by stopping the mechanisms etc., instead of focusing solely on certain treatments such as cardiac rehabilitation. Medications can be created to hinder the mechanisms directly which is more effective than slowing down the cognitive impairments *via* the overt symptoms. Moreover, prompt detection of CI will bring about better clinical outcomes in HF patients.

## Data Availability

The original contributions presented in the study are included in the article/**Supplementary Material**, further inquiries can be directed to the corresponding author.

## Supplementary material

Supplementary Table 1 PRISMA checklist.

Supplementary Table 2 Search strategy.

Supplementary Table 3 Screening tool.

Supplementary Table 4 Critical appraisal checklists.

Supplementary Table 5 Data extraction forms.

Supplementary Table 6 Summary of quality appraisal.
