# Supplementary material for: The pathophysiology of cognitive impairment in individuals with heart failure: a systematic review
Source: Front Cardiovasc Med. 2023 May 23;10:1181979. doi: 10.3389/fcvm.2023.1181979 (PMC10242665; doi:10.3389/fcvm.2023.1181979)
Supplement: Supplementary Table 2 — Search strategy. [file Table2.docx]

Supplementary Material

Appendix B

# Search Strategy Concepts and Keywords

|  | **Concept 1: Population** | **Concept 2: Exposure** | **Additional concept** |
| --- | --- | --- | --- |
| **Key concepts** | Individuals with heart failure | Cognitive impairment | Pathophysiology |
| **Free text terms / natural language terms** | heart failure OR cardiac failure OR congestive heart failure OR reduced ejection fraction OR myocardial dysfunction OR systolic dysfunction OR diastolic dysfunction OR Heart Decompensation OR Myocardial Failure OR cardiac decompensation | cognitive impairment* OR cognitive problem* OR cognitive dysregulation OR cognitive dysfunction OR cognitive disorder* OR cognitive defect* OR neurodegenerat* OR dement* OR cognitive derangement OR cognitive decline OR Mental Deterioration OR Mild Neurocognitive Disorder* OR Alzheimer* OR brain impairment* OR brain problem* OR brain dysregulation OR brain dysfunction OR brain disorder* OR brain defect* OR brain decline OR brain derangement | caus* OR pathophysiology OR reason* OR heart-brain connection* heart-brain OR etiology OR aetiology OR physiopathology |

# PubMed Search Strategy

| **PubMed**  **Filters:**  2013-2022 | #1 | (“Heart Failure”[Mesh] OR (“heart failure”[Title/Abstract] OR “cardiac failure”[Title/Abstract] OR “reduced ejection fraction”[Title/Abstract] OR “myocardial dysfunction”[Title/Abstract] OR “systolic dysfunction”[Title/Abstract] OR “diastolic dysfunction”[Title/Abstract] OR “heart decompensation”[Title/Abstract] OR “myocardial failure”[Title/Abstract] OR “cardiac decompensation”[Title/Abstract] OR “congestive heart failure”[Title/Abstract])) AND (“Cognitive Dysfunction”[Mesh] OR “Dementia”[Mesh] OR (“cognitive impairment*”[Title/Abstract] OR “cognitive problem*”[Title/Abstract] OR “cognitive dysregulation”[Title/Abstract] OR “cognitive dysfunction”[Title/Abstract] OR “cognitive disorder*”[Title/Abstract] OR “cognitive defect*”[Title/Abstract] OR neurodegenerat*[Title/Abstract] OR dement*[Title/Abstract] OR “cognitive derangement”[Title/Abstract] OR “cognitive decline”[Title/Abstract] OR “mental deterioration”[Title/Abstract] OR “mild neurocognitive disorder*”[Title/Abstract] OR Alzheimer*[Title/Abstract] OR “brain impairment*”[Title/Abstract] OR “brain problem*”[Title/Abstract] OR “brain dysregulation”[Title/Abstract] OR “brain dysfunction”[Title/Abstract] OR “brain disorder*”[Title/Abstract] OR “brain defect*”[Title/Abstract] OR “brain decline”[Title/Abstract] OR “brain derangement”[Title/Abstract])) AND (“Causality”[Mesh] OR “physiopathology”[Subheading] OR “etiology”[Subheading] OR (caus*[Title/Abstract] OR pathophysiology[Title/Abstract] OR physiopathology[Title/Abstract] OR reason*[Title/Abstract] OR “heart-brain connection*”[Title/Abstract] OR “heart-brain”[Title/Abstract] OR etiology[Title/Abstract] OR aetiology[Title/Abstract])) |
| --- | --- | --- |

# The Cochrane Library Search Strategy

| **The Cochrane Library**  **Filters:**  Jan 2013- Dec 2022 (Cochrane Library publication date), Word variations | #1  #2  #3  #4  #5  #6  #7  #8  #9  #10  #11  #12  #13 | MeSH descriptor: [Cognitive Dysfunction] explode all trees  MeSH descriptor: [Dementia] explode all trees  MeSH descriptor: [Neurocognitive disorders] explode all trees  MeSH descriptor: [Heart Failure] explode all trees  MeSH descriptor: [Causality] explode all trees  Any MeSH descriptor in all MeSH products and with qualifier(s): [physiopathology - PP]  Any MeSH descriptor in all MeSH products and with qualifier(s): [etiology - ET]  (caus*):ti,ab,kw OR (pathophysiology):ti,ab,kw OR (reason*):ti,ab,kw OR (“Heart-brain connection*”):ti,ab,kw  ("heart-brain"):ti,ab,kw OR (etiology):ti,ab,kw OR (aetiology):ti,ab,kw OR (physiopathology):ti,ab,kw  ((brain or cognitive) NEAR/3 (impairment* or problem* or dysregulation or dysfunction or disorder* or defect* or decline or derangement)):ti,ab,kw  (neurodegenerat*):ti,ab,kw OR (dement*):ti,ab,kw OR (“mental deterioration”):ti,ab,kw OR (“mild neurocognitive disorder*”):ti,ab,kw OR (alzheimer*):ti,ab,kw  (reduced NEAR/2 (ejection NEXT fraction)):ti,ab,kw OR ((heart or cardiac) NEAR/3 decompensation):ti,ab,kw OR ((heart or cardiac or myocardial) NEXT failure):ti,ab,kw OR ((myocardial or systolic or diastolic) NEXT dysfunction):ti,ab,kw OR (“congestive heart failure”):ti,ab,kw  (#1 OR #2 OR #3 OR #10 OR #11) AND (#4 OR #12) AND (#5 OR #6 OR #7 OR #8 OR #9) |
| --- | --- | --- |

# Embase Search Strategy

| **Embase**  **Filters:**  2013-2022 | #1  #2  #3  #4  #5  #6 | (caus*:ab,ti OR pathophysiology:ab,ti OR reason*:ab,ti OR 'heart-brain connection*':ab,ti OR 'heart-brain':ab,ti OR physiopathology:ab,ti OR etiology:ab,ti OR aetiology:ab,ti) AND [2013-2022]/py  OR  'pathophysiology'/exp OR ‘etiology’/exp  AND  'cognitive defect'/exp OR 'dementia'/exp OR 'mental deterioration'/exp OR ‘brain dysfunction’/exp  OR  ('cognitive NEAR/3 impairment*':ab,ti OR 'cognitive NEAR/3 problem*':ab,ti OR 'cognitive NEAR/3 dysregulation':ab,ti OR 'cognitive NEAR/3 dysfunction':ab,ti OR 'cognitive NEAR/3 disorder*':ab,ti OR 'cognitive NEAR/3 defect*':ab,ti OR neurodegenerat*:ab,ti OR dement*:ab,ti OR 'cognitive NEAR/3 derangement':ab,ti OR 'cognitive NEAR/3 decline':ab,ti OR 'mental deterioration':ab,ti OR 'mild neurocognitive disorder*':ab,ti OR alzheimer*:ab,ti OR ‘brain NEAR/3 impairment*’:ab,ti OR ‘brain NEAR/3 problem*’:ab,ti OR ‘brain NEAR/3 dysregulation’:ab,ti OR ‘brain NEAR/3 dysfunction’:ab,ti OR ‘brain NEAR/3 disorder*’:ab,ti OR ‘brain NEAR/3 defect*’ OR ‘brain NEAR/3 decline’:ab,ti OR ‘brain NEAR/3 derangement’:ab,ti) AND [2013-2022]/py  AND  'congestive heart failure'/exp OR 'systolic dysfunction'/exp OR 'diastolic dysfunction'/exp OR 'systolic heart failure'/exp OR 'diastolic heart failure'/exp OR 'heart failure'/exp  OR  ('cardiac failure':ab,ti OR ‘myocardial failure’:ab,ti OR 'reduced NEAR/2 (ejection NEXT/1 fraction)':ab,ti OR 'myocardial dysfunction':ab,ti OR 'systolic dysfunction':ab,ti OR 'diastolic dysfunction':ab,ti OR 'heart failure':ab,ti OR ‘heart NEAR/3 decompensation’:ab,ti OR ‘cardiac NEAR/3 decompensation’:ab,ti OR ‘congestive heart failure’:ab,ti) AND [2013-2022]/py |
| --- | --- | --- |

# CINAHL Search Strategy

| **CINAHL**  **Filters:**  January 2013- December 2022, apply related words, apply equivalent subjects | #1  #2 | Title:  ((MH "Physiopathology") OR caus* OR pathophysiology OR reason* OR “heart-brain connection*” OR “heart-brain” OR physiopathology OR etiology OR aetiology) AND ((MH "Mild Cognitive Impairment") OR  “cognitive N3 impairment*” OR “cognitive N3 problem*” OR “cognitive N3 dysregulation” OR “cognitive N3 dysfunction” OR “cognitive N3 disorder*” OR “cognitive N3 defect*” OR neurodegenerat* OR dement* OR “cognitive N3 derangement” OR “cognitive N3 decline” OR “mental deterioration” OR “mild neurocognitive disorder*” OR Alzheimer* OR “brain impairment*” OR “brain N3 problem*” OR “brain N3 dysregulation” OR “brain N3 dysfunction” OR “brain N3 disorder*” OR “brain N3 defect*” OR “brain N3 decline” OR “brain N3 derangement”) AND ((MH "Heart Failure+") OR “heart failure” OR “cardiac failure” OR “reduced N2 (ejection W1 fraction)” OR “myocardial dysfunction” OR “systolic dysfunction” OR “diastolic dysfunction” OR “heart N3 decompensation” OR “myocardial failure” OR “cardiac N3 decompensation” OR “congestive heart failure”)  OR  Abstract:  ((MH "Physiopathology") OR caus* OR pathophysiology OR reason* OR “heart-brain connection*” OR “heart-brain” OR physiopathology OR etiology OR aetiology) AND ((MH "Mild Cognitive Impairment") OR  “cognitive N3 impairment*” OR “cognitive N3 problem*” OR “cognitive N3 dysregulation” OR “cognitive N3 dysfunction” OR “cognitive N3 disorder*” OR “cognitive N3 defect*” OR neurodegenerat* OR dement* OR “cognitive N3 derangement” OR “cognitive N3 decline” OR “mental deterioration” OR “mild neurocognitive disorder*” OR Alzheimer* OR “brain impairment*” OR “brain N3 problem*” OR “brain N3 dysregulation” OR “brain N3 dysfunction” OR “brain N3 disorder*” OR “brain N3 defect*” OR “brain N3 decline” OR “brain N3 derangement”) AND ((MH "Heart Failure+") OR “heart failure” OR “cardiac failure” OR “reduced N2 (ejection W1 fraction)” OR “myocardial dysfunction” OR “systolic dysfunction” OR “diastolic dysfunction” OR “heart N3 decompensation” OR “myocardial failure” OR “cardiac N3 decompensation” OR “congestive heart failure”) |
| --- | --- | --- |

# PsycINFO Search Strategy

| **PsycINFO (OVID)**  **Filters:**  2013-2022 | #1  #2  #3  #4  #5  #6 | heart disorders/  OR  (heart failure or cardiac failure or (reduced adj2 (ejection fraction)) or myocardial dysfunction or systolic dysfunction or diastolic dysfunction or (heart adj3 decompensation) or myocardial failure or (cardiac adj3 decompensation) or congestive heart failure).ab,ti.  AND  exp Cognitive Impairment/ OR exp brain disorders/ OR exp dementia/  OR  ((cognitive adj3 impairment*) or (cognitive adj3 problem*) or (cognitive adj3 dysregulation) or (cognitive adj3 dysfunction) or (cognitive adj3 disorder*) or (cognitive adj3 defect*) or neurodegenerat* or dement* or (cognitive adj3 derangement) or (cognitive adj3 decline) or mental deterioration or mild neurocognitive disorder* or Alzheimer* or (brain adj3 impairment*) or (brain adj3 problem*) or (brain adj3 dysregulation) or (brain adj3 dysfunction) or (brain adj3 disorder*) or (brain adj3 defect*) or (brain adj3 decline) or (brain adj3 derangement)).ab,ti.  AND  exp Pathophysiology/ OR exp etiology/  OR  (caus* or pathophysiology or reason* or heart-brain connection* or heart-brain or physiopathology or etiology or aetiology).ab,ti. |
| --- | --- | --- |

# Scopus Search Strategy

| **Scopus**  **Filters:**  2013-2022 | #1 | TITLE-ABS-KEY ( ( caus* OR pathophysiology OR reason* OR "heart-brain connection” OR "heart-brain" OR physiopathology OR etiology OR aetiology) AND ( ("cognitive W/3 impairment*") OR ("cognitive W/3 problem*") OR ("cognitive W/3 dysregulation") OR ("cognitive W/3 dysfunction") OR ("cognitive W/3 disorder*") OR ("cognitive W/3 defect*") OR neurodegenerat* OR dement* OR ("cognitive W/3 derangement") OR ("cognitive W/3 decline") OR "mental deterioration" OR "mild neurocognitive disorder*" OR alzheimer* OR ("brain W/3 impairment*") OR ("brain W/3 problem*") OR ("brain W/3 dysregulation") OR ("brain W/3 dysfunction") OR ("brain W/3 disorder*") OR ("brain W/3 defect*") OR ("brain W/3 derangement") OR ("brain W/3 decline")) AND ( "heart failure" OR "cardiac failure" OR ("reduced W/2 (ejection PRE/1 fraction)") OR "myocardial dysfunction" OR "systolic dysfunction" OR "diastolic dysfunction" OR ("heart W/3 decompensation") OR "myocardial failure" OR ("cardiac W/3 decompensation") OR “congestive heart failure”) ) AND ( LIMIT-TO ( PUBYEAR , 2022 ) OR LIMIT-TO ( PUBYEAR , 2021 ) OR LIMIT-TO ( PUBYEAR , 2020 ) OR LIMIT-TO ( PUBYEAR , 2019 ) OR LIMIT-TO ( PUBYEAR , 2018 ) OR LIMIT-TO ( PUBYEAR , 2017 ) OR LIMIT-TO ( PUBYEAR , 2016 ) OR LIMIT-TO ( PUBYEAR , 2015 ) OR LIMIT-TO ( PUBYEAR , 2014 ) OR LIMIT-TO ( PUBYEAR , 2013 ) ) |
| --- | --- | --- |

# Web of Science Search Strategy

| **Web of Science**  **Filters:**  1 January 2013-31 December 2022 | #1 | Topic:  (“heart failure” OR “cardiac failure” OR “reduced ejection fraction” OR “myocardial dysfunction” OR “systolic dysfunction” OR “diastolic dysfunction” OR “heart decompensation” OR “myocardial failure” OR “cardiac decompensation” OR “congestive heart failure”) AND ( “cognitive impairment*” OR “cognitive problem*” OR “cognitive dysregulation” OR “cognitive dysfunction” OR “cognitive disorder*” OR “cognitive defect*” OR neurodegenerat* OR dement* OR “cognitive derangement” OR “cognitive decline” OR “mental deterioration” OR “mild neurocognitive disorder*” OR Alzheimer* OR “brain impairment*” OR “brain problem*” OR “brain dysregulation” OR “brain dysfunction” OR “brain disorder*” OR “brain defect*” OR “brain decline” OR “brain derangement”) AND (caus* OR pathophysiology OR reason* OR “heart-brain connection*” OR “heart-brain" OR physiopathology OR etiology OR aetiology) |
| --- | --- | --- |

# ProQuest Dissertations and Theses Global (Social Science Premium Collection) Search Strategy

| **ProQuest Dissertations and Theses Global (Social Science Premium Collection)**  **Filters:**  1 January 2013-31 December 2022 | #1 | ((MAINSUBJECT.EXACT("Congestive heart failure") OR MAINSUBJECT.EXACT("Heart failure")) OR (tiab(“heart failure”) OR tiab(“cardiac failure”) OR tiab(“reduced ejection fraction”) OR tiab(“myocardial dysfunction”) OR tiab(“systolic dysfunction”) OR tiab(“diastolic dysfunction”) OR tiab(“heart decompensation”) OR tiab(“myocardial failure”) OR tiab(“cardiac decompensation”) OR tiab(“congestive heart failure”))) AND ((MAINSUBJECT.EXACT("Pathophysiology") OR MAINSUBJECT.EXACT("Etiology")) OR (tiab(caus*) OR tiab(pathophysiology) OR tiab(reason*) OR tiab(“heart-brain connection*”) OR tiab(“heart-brain”) OR tiab(physiopathology) OR tiab(etiology) OR tiab(aetiology))) AND ((MAINSUBJECT.EXACT("Dementia")) OR (tiab(“cognitive impairment*”) OR tiab(“cognitive problem*”) OR tiab(“cognitive dysregulation”) OR tiab(“cognitive dysfunction”) OR tiab(“cognitive disorder*”) OR tiab(“cognitive defect*”) OR tiab(neurodegenerat*) OR tiab(dement*) OR tiab(“cognitive derangement”) OR tiab(“cognitive decline”) OR tiab(“mental deterioration”) OR tiab(“mild neurocognitive disorder*”) OR tiab(“Alzheimer*”) OR tiab(“brain impairment*”) OR tiab(“brain problem*”) OR tiab(“brain dysregulation”) OR tiab(“brain dysfunction”) OR tiab(“brain disorder*”) OR tiab(“brain defect*”) OR tiab(“brain decline”) OR tiab(“brain derangement”))) |
| --- | --- | --- |

# Grey Literature: Mednar Search Strategy

| **Mednar**  **Filters:**  2013-2022 | #1 | Full Record:  ( caus* OR pathophysiology OR reason* OR "heart-brain connection*" OR "heart-brain" OR physiopathology OR etiology OR aetiology) AND ( ("cognitive AROUND (3) impairment*") OR ("cognitive AROUND (3) problem*") OR ("cognitive AROUND (3) dysregulation") OR ("cognitive AROUND (3) dysfunction") OR ("cognitive AROUND (3) disorder*") OR ("cognitive AROUND (3) defect*") OR neurodegenerat* OR dement* OR ("cognitive AROUND (3) derangement") OR ("cognitive AROUND (3) decline") OR "mental deterioration" OR "mild neurocognitive disorder*" OR alzheimer* OR ("brain AROUND (3) impairment*") OR ("brain AROUND (3) problem*") OR ("brain AROUND (3) dysregulation") OR ("brain AROUND (3) dysfunction") OR ("brain AROUND (3) disorder*") OR ("brain AROUND (3) defect*") OR ("brain AROUND (3) derangement") OR ("brain AROUND (3) decline")) AND ( "heart failure" OR "cardiac failure" OR ("reduced AROUND (2) (ejection AROUND (1) fraction)") OR "myocardial dysfunction" OR "systolic dysfunction" OR "diastolic dysfunction" OR ("heart AROUND (3) decompensation") OR "myocardial failure" OR “cardiac AROUND (3) decompensation” OR “congestive heart failure” ) |
| --- | --- | --- |

# Grey Literature: ProQuest Dissertations and Theses Global Search Strategy

| **ProQuest Dissertations and Theses Global**  **Filters:**  2013-2022 | #1 | ( caus* OR pathophysiology OR reason* OR "heart-brain connection*" OR "heart brain connection*" OR "heart and brain" OR "heart brain" OR physiopathology OR etiology OR aetiology) AND ( ("cognitive impairment*") OR ("cognitive problem*") OR ("cognitive dysregulation") OR ("cognitive dysfunction") OR ("cognitive disorder*") OR ("cognitive defect*") OR neurodegenerat* OR dement* OR ("cognitive derangement") OR ("cognitive decline") OR "mental deterioration" OR "mild neurocognitive disorder*" OR alzheimer* OR ("brain impairment*") OR ("brain problem*") OR ("brain dysregulation") OR ("brain dysfunction") OR ("brain disorder*") OR ("brain defect*") OR ("brain derangement") OR ("brain decline")) AND ( "heart failure" OR "cardiac failure" OR "reduced ejection fraction" OR "myocardial dysfunction" OR "systolic dysfunction" OR "diastolic dysfunction" OR ("heart decompensation") OR "myocardial failure" ) |
| --- | --- | --- |
