# Supplementary material for: The pathophysiology of cognitive impairment in individuals with heart failure: a systematic review
Source: Front Cardiovasc Med. 2023 May 23;10:1181979. doi: 10.3389/fcvm.2023.1181979 (PMC10242665; doi:10.3389/fcvm.2023.1181979)
Supplement: Supplementary Table 3 — Screening tool. [file Table3.docx]

Supplementary Material

Appendix C

# Screening Tool for Citation, Title and Abstract Screening

Does the citation indicate publication in or after 2013?

Yes: continue screening

No: stop screening

Does the title or abstract indicate that it is a study in relation to heart failure and cognitive impairment? - “not HF and CI” *

Yes: continue screening

No: stop screening

Does the title or abstract indicate that this is a primary study? - “non primary study” *

Yes: continue screening

No: stop screening

Does the title or abstract use English or Chinese? - “language” *

Yes: continue screening

No: stop screening

Does the title or abstract indicate that this is not an intervention type of study? – “intervention type study” *

Yes: continue screening

No: stop screening

Does the abstract talk about the mechanisms/pathophysiology of cognitive impairment in heart failure? – “not related to pathophysiology” *

Yes or Unsure/Unclear: continue screening

No: stop screening

Does the abstract indicate that the study uses a quantitative design?

Yes: continue screening

No: stop screening

# Screening Tool for Full Text Screening

8. Does the full text fit the eligibility criteria of the participants? –“HF occurred after CI” *

Yes: continue screening

No: stop screening

9. Is there access to the full text? –“no access” *

Yes: continue screening

No: stop screening

**Decision**: Should this article be included?

a. Yes, all 9 screening questions answered Yes or Unclear

b. No, at least one answers definitely “No”

* The labels included in the Rayyan screening tool for exclusion of the studies during the screening stage
