# Supplementary material for: The pathophysiology of cognitive impairment in individuals with heart failure: a systematic review
Source: Front Cardiovasc Med. 2023 May 23;10:1181979. doi: 10.3389/fcvm.2023.1181979 (PMC10242665; doi:10.3389/fcvm.2023.1181979)
Supplement: Supplementary Table 5 — Data extraction forms. [file Table5.docx]

Supplementary Material

Appendix E

# Data Extraction Form (Human Study)

| **General Study Details** | |
| --- | --- |
| Title |  |
| Record Number |  |
| Author(s) |  |
| Year of Publication |  |
| Journal |  |
| Study design |  |
| Aim |  |
| **Participant** **characteristics** | |
| Total number of participants |  |
| Age range |  |
| Gender |  |
| Type of HF: HFpEF / HFrEF |  |
| Type of HF: Ischemic / Non-ischemic etiology |  |
| Type of HF: Acute / Chronic HF |  |
| Cardiac Function Grade |  |
| **Methods** | |
| Recruitment methods |  |
| Type of tools used |  |
| Study duration |  |
| **Study result(s) / outcome(s)** | |
| Outcome & the relevant measurements |  |
| Conclusion(s) made |  |
| **Data Analysis methods** | |
| Statistical technique/analysis |  |
| **Others** | |
|  | |
| **Reviewer’s comments** | |
|  | |

# Data Extraction Form (Animal Study)

| **General Study Details** | |
| --- | --- |
| Title |  |
| Record Number |  |
| Author(s) |  |
| Year of Publication |  |
| Journal |  |
| Study design |  |
| Aim |  |
| **Participant** **characteristics** | |
| Total number of participants |  |
| Gender |  |
| **Methods** | |
| Setting |  |
| Preparation done |  |
| Type of tools/tests used |  |
| Study duration |  |
| **Study result(s) / outcome(s)** | |
| Outcomes & the relevant measurements |  |
| Conclusion(s) made |  |
| **Data Analysis methods** | |
| Statistical technique/analysis |  |
| **Others** | |
|  | |
| **Reviewer’s comments** | |
|  | |
