# Supplementary material for: The pathophysiology of cognitive impairment in individuals with heart failure: a systematic review
Source: Front Cardiovasc Med. 2023 May 23;10:1181979. doi: 10.3389/fcvm.2023.1181979 (PMC10242665; doi:10.3389/fcvm.2023.1181979)
Supplement: Supplementary Table 6 — Summary of quality appraisal. [file Table6.docx]

Supplementary Material

Appendix F

# Summary of the Critical Appraisal of Case-Control Studies


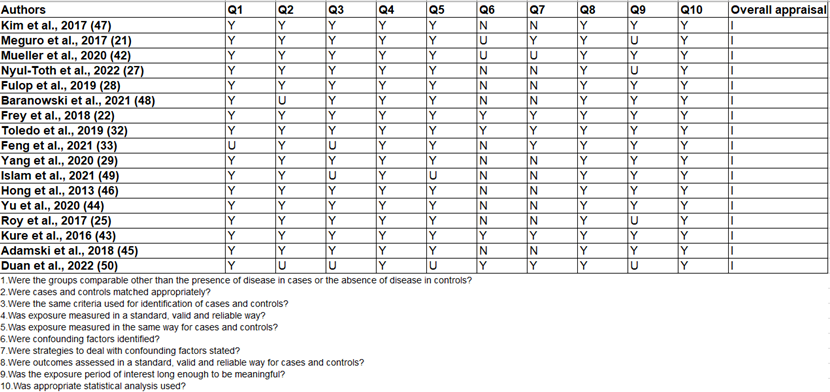


# Summary of the Critical Appraisal of Cross-Sectional Studies


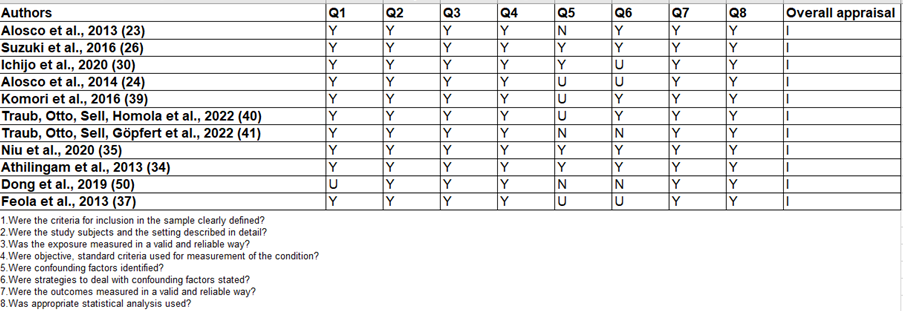


# Summary of the Critical Appraisal of Cohort Studies


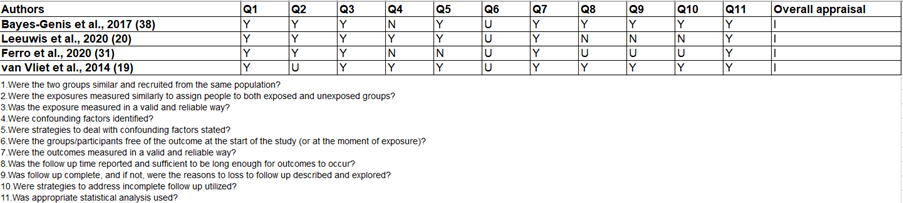


**Legend:** Y-Yes, N-No, U-Unclear, I-Include
